# TBI Server: A Web Server for Predicting Ion Effects in RNA Folding

**DOI:** 10.1371/journal.pone.0119705

**Published:** 2015-03-23

**Authors:** Yuhong Zhu, Zhaojian He, Shi-Jie Chen

**Affiliations:** 1 Department of Physics, Department of Biochemistry, and Informatics Institute, University of Missouri, Columbia, MO 65211, USA; Department of Physics, Hangzhou Normal University, Hangzhou, Zhejiang 310036, China; 2 Department of Physics, Department of Biochemistry, and Informatics Institute, University of Missouri, Columbia, MO 65211, USA; 3 Department of Physics, Department of Biochemistry, and Informatics Institute, University of Missouri, Columbia, MO 65211, USA; Hong Kong University of Science and Technology, HONG KONG

## Abstract

**Background:**

Metal ions play a critical role in the stabilization of RNA structures. Therefore, accurate prediction of the ion effects in RNA folding can have a far-reaching impact on our understanding of RNA structure and function. Multivalent ions, especially Mg^2+^, are essential for RNA tertiary structure formation. These ions can possibly become strongly correlated in the close vicinity of RNA surface. Most of the currently available software packages, which have widespread success in predicting ion effects in biomolecular systems, however, do not explicitly account for the ion correlation effect. Therefore, it is important to develop a software package/web server for the prediction of ion electrostatics in RNA folding by including ion correlation effects.

**Results:**

The TBI web server http://rna.physics.missouri.edu/tbi_index.html provides predictions for the total electrostatic free energy, the different free energy components, and the mean number and the most probable distributions of the bound ions. A novel feature of the TBI server is its ability to account for ion correlation and ion distribution fluctuation effects.

**Conclusions:**

By accounting for the ion correlation and fluctuation effects, the TBI server is a unique online tool for computing ion-mediated electrostatic properties for given RNA structures. The results can provide important data for in-depth analysis for ion effects in RNA folding including the ion-dependence of folding stability, ion uptake in the folding process, and the interplay between the different energetic components.

## Introduction

Because RNA backbone is highly negatively charged, the folding of RNA requires counterions to neutralize the backbone charge and to reduce Coulomb repulsion. As a result, RNA folding is sensitive to the ionic condition, such as ion type, size, valence and concentration [1–12]. The interaction between counterions (metal ions) and RNA plays a critical role in RNA folding, including the structure and the folding stability and folding kinetics [13–15]. Accurate evaluation of the ion electrostatic effect is essential for the prediction of RNA folding.

One of the challenges in modeling the ion effects is how to treat the potentially important ion correlation and fluctuation effects. Coulomb interaction is a long-range force. As a result, the electric force acting on an ion is a function not only of the its own coordinates but also of the simultaneous positions of the other ions. In an ionic solution, ions have a strong tendency to accumulate in the close vicinity of (the negatively charged) RNA. The ions could reach high local density which leads to ion correlation. One of the resultant effects from ion correlation is the coupling between the ion binding events at the different sites. Such a coupling effect is stronger for multivalent ions than monovalent ions due to their higher charges. Motivated by the importance to treat ion correlation effects, especially for multivalent ions such as Mg^2+^ ions, which are essential for the stabilization of RNA tertiary structure, we developed the Tightly Bound Ion (TBI) model [16–20]. To treat the correlation effect inevitably requires the consideration of the ensemble of discrete many-ion distributions instead of a mean-field distribution. Thus, the TBI model can also account for the fluctuations in ion distribution.

The TBI model is a theory for predicting ion-dependent RNA folding stability [16–20]. The model was first reported in 2005 [16] and further developed in 2008 [17] with explicit inclusion of the solvent polarization effect through the Generalized Born model. In 2012 [20], with an energy landscape-guided approach for the sampling of ion distribution, the model undergoes a significant improvement with a drastically enhanced computational efficiency. The enhanced version of the TBI allows us to treat RNAs of sequences longer than 80 nucleotides. For example, with enhanced version of the model, the computational time of a Tetraloop-receptor system of 81 nucleotides is about 30–80 minutes for the different ionic conditions [21]. Tests of the TBI predictions against the experimental data for ion binding properties and ion-dependent folding stabilities (Table 1.) [17–19, 22] suggested that the TBI model may be reliable for predicting ion effects in RNA folding.

**Table 1:** Comparison between the TBI and the Poisson-Boltzmann predictions and test against experimental data.

RNA or DNA	Comparison of parameters	Reference	The average error for BP	The average error for TBI
Three DNA helices	Folding free energy	Fig. 2d [22]	1.2 kcal/mol	0.1 kcal/mol
Two DNA helices	Melting temperature	Fig. 5 [23]	5.4°C	1.0°C
Two RNA helices	Ion binding Fraction	Fig. 2a [23]	0.11	0.06
24bp B-DNA helix	Ion binding Fraction	Fig. 2a [24]	0.08	0.01
40bp A-RNA helix	Ion binding Fraction	Fig. 2c [24]	0.15	0.04
40bp B-DNA helix	Ion binding Fraction	Fig. 2e [24]	0.12	0.03
BWYV pseudoknot RNA	Ion binding Fraction	Fig. 3a [24]	0.05	0.03
58-nt rRNA	Ion binding Fraction	Fig. 3c [24]	0.10	0.03
Yeast tRNA^*Phe*^	Ion binding Fraction	Fig. 3e [24]	0.06	0.03

## Methods

### The Tightly Bound Ion (TBI) model

We classify the ions into two types according to their Coulomb correlation strengths: The tightly bound ions (of strong correlation) and the diffusive ions (of weak correlation). The region (a thin layer around the RNA surface) where the tightly bound ions are distributed is called the tightly bound region. For an *N*-nt RNA, the tightly bound region can be divided into *N* cells, each around a phosphate. For the tightly bound ions, we sample the discrete modes of ion distribution. Here a mode is defined by the number of bound ions in the cells. Through enumeration of the discrete ion binding modes and evaluation of the multi-ion electrostatic energy for each mode, the TBI model accounts for the correlation between the bound ions and the fluctuation of ion distributions [16–19, 23, 24].

For a given ion binding mode *M*, ions are allowed to move inside the respective cells. By sampling the coordinates of the tightly bound ions within their respective cells (*dR*_i_ below), we calculate the partition function *Z*_M_ of the system:

$$\begin{array}{c} Z_{M}=Z^{\left(id\right)}{\left(\frac{N_{Z}}{V}\right)}^{N_{b}}\left(\int \prod_{i=1}^{N_{b}}dR_{i}\right)e^{-\Delta G_{M}/k_{B}T}, \end{array}$$ (1)

where *Z* ^(*id*)^ is the partition function for the uniform ion solution (without the RNA), *N*_Z_ is the total number of z-valent counterions and *V* is the volume of the solution, *N*_b_ and $\int \Pi_{\text{i}=1}^{N_{\text{b}}}dR_{\text{i}}$ are the number and the volume integral for the tightly bound ions, respectively, and Δ*G*_*M*_ is the free energy of the system for mode *M*.

### Electrostatic free energy for each mode

The electrostatic energy for the charges inside the tightly bound region is computed as the sum of the self-energy Δ*U*_self_, the polarization energy Δ*U*_pol_, and the Coulomb energy Δ*U*_ele_:

$$\begin{array}{ccc} \Delta U_{\mathrm{self}} & = & \frac{1}{2}\left(\frac{1}{\epsilon_{w}}-\frac{1}{\epsilon_{\mathrm{in}}}\right)\;\sum_{p}\frac{q_{p}^{2}}{B_{i}}+\left(\frac{1}{\epsilon_{w}}-\frac{1}{\epsilon_{\mathrm{in}}}\right)\;\sum_{i}\frac{q_{i}^{2}}{2}\left(\frac{1}{B_{i}}-\frac{1}{B_{i}^{0}}\right)\\ \Delta U_{\mathrm{pol}} & = & \left(\frac{1}{\epsilon_{w}}-\frac{1}{\epsilon_{\mathrm{in}}}\right)\sum_{m<n}\frac{q_{m}q_{n}}{\sqrt{r_{\mathrm{mn}}^{2}+B_{m}B_{n}\exp\left(-\frac{r_{\mathrm{mn}}^{2}}{4B_{m}B_{n}}\right)}}\\ \Delta U_{\mathrm{ele}} & = & \sum_{m<n}\frac{q_{m}q_{n}}{\epsilon_{\mathrm{in}}r_{\mathrm{mn}}} \end{array}$$ (2)

where ∑_*p*_, ∑_i_, ∑_mn_ are the summations over the phosphates, tightly bound ions, and both, respectively, and *q*_*p*_, *q*_*i*_, *q*_*m*_ *q*_*n*_ are the respective charges. *r*_mn_ is the distance between *m* and *n*, *B*_*x*_ is the Born radius of *x*, $B_{i}^{0}$ is the radius of the hydrated ion, and *ϵ*_in_ (∼ 12) and *ϵ*_w_ (∼ 78 at room temperature) are the dielectric constants of RNA and water, respectively. *ϵ*_w_ is given by

$$\begin{array}{c} \epsilon_{w}\left(T\right)=87.740-\left(0.4008\right)\;T+\left(9.398\times 10^{-4}\right)\;T^{2}-\left(1.41\times 10^{-6}\right)\;T^{3}, \end{array}$$ (3)

where *T* is the temperature in Celsius.

The free energy of the diffusive ions is calculated as the sum of the enthalpic Δ*U*_d_ and entropic −*T*Δ*S*_d_ terms:

$$\begin{array}{ccc} \Delta U_{d} & = & \frac{1}{2}\int \sum_{\alpha }c_{\alpha }\left(r\right)z_{\alpha }q\left[\Psi \left(r\right)+\Psi^{'}\left(r\right)\right]d^{3}r\\ \Delta S_{d} & = & -k_{B}\int \sum_{\alpha }\left[c_{\alpha }\left(r\right)\ln\frac{c_{\alpha }\left(r\right)}{c_{\alpha }^{0}}-c_{\alpha }\left(r\right)+c_{\alpha }^{0}\right]\;d^{3}r \end{array}$$ (4)

where *c*_α_ is the concentration of ion species *α*, $c_{\text{α}}^{0}$ is the bulk concentration, Ψ(*r*) and Ψ^′^(*r*) are the electrostatic potentials with and without the diffusive ions, respectively. The electric potentials are determined from the Poisson-Boltzmann equation with the presence of the tightly bound ions.

For a given mode *M*, the ensemble average over the positions of the tightly bound ions, denoted as ⟨…⟩, gives the electrostatic free energy components: Δ*G*_*M*_ = ⟨Δ*U*_self_ + Δ*U*_pol_ + Δ*U*_ele_ + Δ*U*_d_ − *T*Δ*S*_d_⟩.

### Free energy components

Averaging over all the tightly bound ion distribution modes gives the electrostatic free energy of the system:

$$\begin{array}{c} \Delta G_{\mathrm{tot}}=-k_{B}T\ln\sum_{M}\left(Z_{M}/Z^{\left(id\right)}\right). \end{array}$$ (5)

The probability of mode *M* is P_M_ = *e* ^−(Δ*G*_*M*_−Δ*G*_tot_)/*k*_B_*T*^.

The weighted sum over all the modes gives the free energy components:

- Coulomb free energy Δ*E*_ele_ = ∑_*M*_⟨Δ*U*_ele_⟩P_M_
- Polarization free energy Δ*G*_pol_ = ∑_*M*_⟨Δ*U*_pol_⟩P_M_
- Self-polarization free energy Δ*G*_self_ = ∑_*M*_⟨Δ*U*_self_⟩P_M_
- Entropic free energy Δ*G*_*s*_ = Δ*G*_tot_ − (Δ*E*_ele_ + Δ*G*_pol_ + Δ*G*_self_)

Here the entropic free energy Δ*G*_*s*_ includes the entropy of the diffusive ions and the (combinatorial) entropy of the tightly bound ions.

### Binding fraction

The TBI model [23, 24] gives the mean binding fraction of Na^+^ and Mg^2+^ ions on each nucleotide *i*:

$$\begin{array}{ccc} f_{{\mathrm{Mg}}^{2+}}^{\left(i\right)} & = & {\bar{f}}_{b}^{\left(i\right)}+\frac{1}{N}\int \left[c_{{\mathrm{Mg}}^{2+}}\left(r\right)-c_{{\mathrm{Mg}}^{2+}}^{0}\right]\;d^{3}r \end{array}$$ (6)

$$\begin{array}{ccc} f_{{\mathrm{Na}}^{+}} & = & \frac{1}{N}\int \left[c_{{\mathrm{Na}}^{+}}\left(r\right)-c_{{\mathrm{Na}}^{+}}^{0}\right]\;d^{3}r \end{array}$$ (7)

where *N* is the number of the nucleotides in RNA and ${\bar{f}}_{b}^{\left(i\right)}$ is the average binding fraction of the tightly bound Mg^2+^ ions on nucleotide *i*:

$$\begin{array}{c} {\bar{f}}_{b}^{\left(i\right)}=\sum_{M}N_{b}^{\left(i\right)}\;P_{M} \end{array}$$ (8)

where $N_{b}^{\left(i\right)}$ is the number of the bound ions on the *i* ^*th*^ nucleotide in ion binding mode *M*. We note the different expressions above for Mg^2+^ and Na^+^ ions. This is because the monovalent ions (Na^+^) are usually weakly correlated and do not exist in the form of tightly bound ions.

## Results

### Input

**Fig 1:**
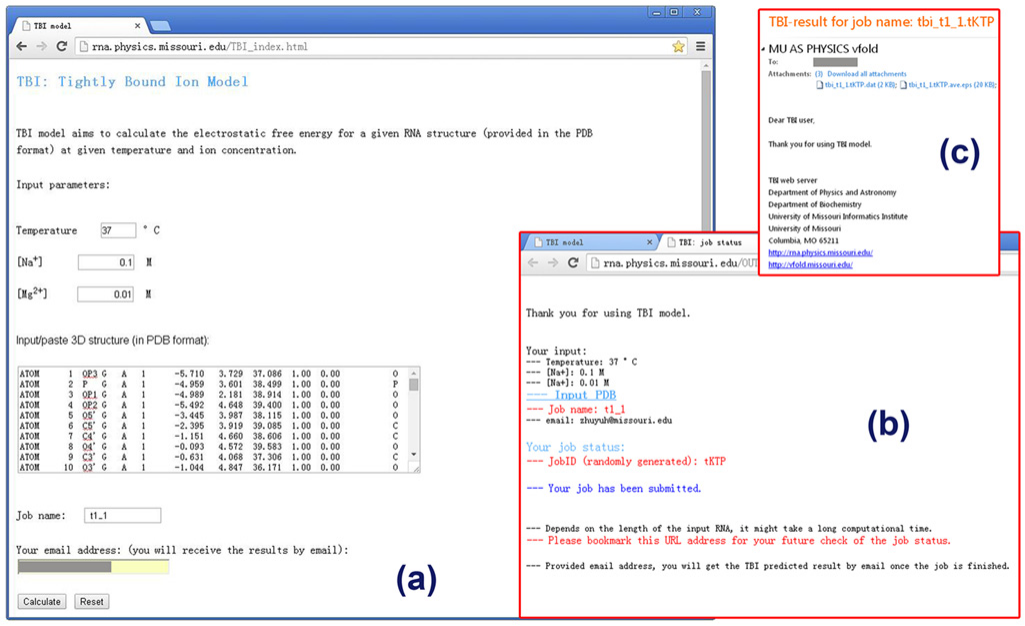
The user interface of the TBI server. (a) the job submission page, (b) the notification page, and (c) the result email.

The TBI server predicts the electrostatic thermodynamic properties for a given RNA structure. The server has a simple input interface (see Fig. 1). The input parameters include the temperature, the monovalent/divalent ion concentrations, and the RNA structure. The current version of the TBI server allows only Na^+^ and Mg^2+^ ions. The server accepts the standard PDB format for the input RNA 3D structure. The user can paste the PDB file into the text window for the input structure. To conveniently identify the submitted jobs, user has the option to define the job names. The user also has the option to retrieve the calculated results either through email (provided by the user) or from a webpage.

For example, to calculate the electrostatic free energy for T2 pseudoknot (PDB code: 2TPK [25], an RNA pseudoknot), we input the parameters as shown in Fig. 1a and the 3D structure of the RNA by pasting the text data of the 2TPK PDB file. We can choose to receive the calculated results through email with the job name and job ID (automatically generated by web server) shown in the subject line (see Fig. 1b)

### Output

**Fig 2:**
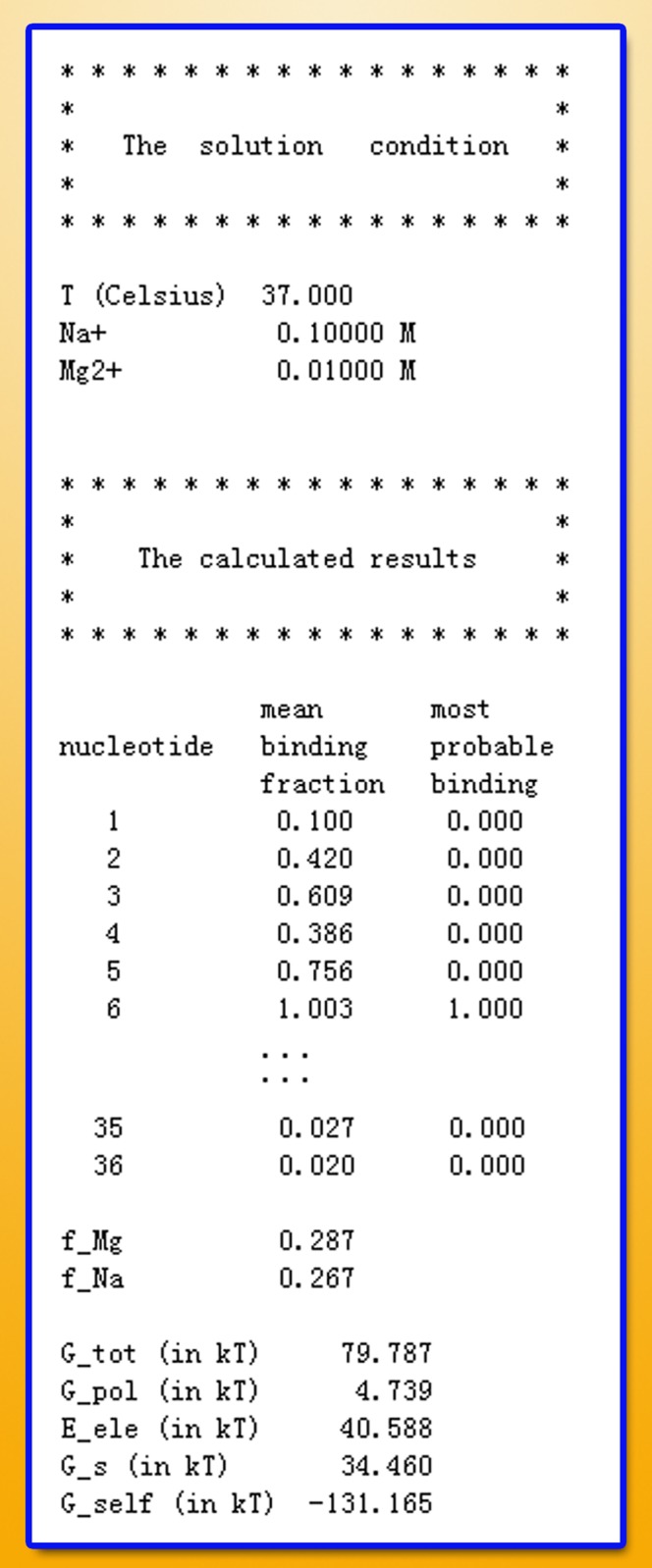
An example for the result text file. The file includes two parts: the solution condition and the calculated results.

The results from the TBI server are presented in three output files, one text file and two figure files (see Fig. 1c). The text file (with filename extension “.dat”) (shown in Fig. 2) shows the solution condition (input parameters) and the numerical results of the predicted fractional bound ions and the free energies.

**Fig 3:**
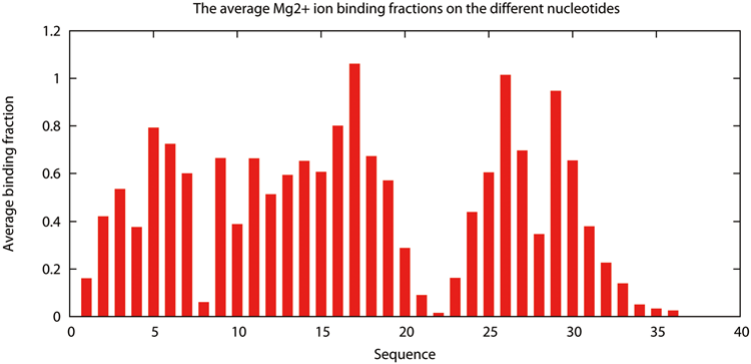
The average binding fraction. The average binding fraction on each nucleotide of the T2 RNA pseudoknot in 10mM Mg^2+^ and 100 mM Na^+^ at *T* = 37°C.

**Fig 4:**
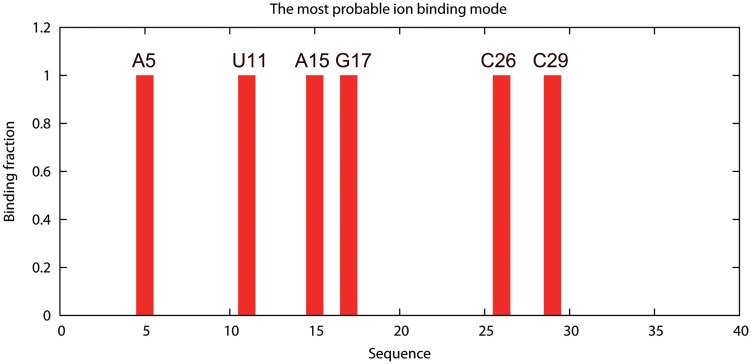
The most probable binding mode for the tightly bound ions. The lowest energy ion binding mode for the T2 RNA pseudoknot at [Mg^2+^] = 10mM, [Na^+^] = 100mM, *T* = 37°C. The most probable binding sites (nucleotides) are denoted with the orange color in the inset.

In the output data file, the first column is the index number of the nucleotides, and the third and the fifth column are the average ion binding fraction for each nucleotide (Equations 6 & 7) and the most probable binding mode (the mode *M* of the lowest free energy Δ*G*_M_) [16], respectively. For example, the number “1” in the fifth column means that the corresponding nucleotide has one tightly bound Mg^2+^ ion in the most probable ion binding mode, while the number “0” means there is no tightly bound Mg^2+^ bound to the nucleotide in the most probable mode. The ion binding properties are shown diagrammatically in the two output figure files, which give the mean (see Fig. 3) and the most probable ion binding modes, respectively (see Fig. 4).

The data file also gives the total electrostatic free energy Δ*G*_tot_ and the free components: the self-polarization energy Δ*G*_self_, the polarization energy Δ*G*_pol_, the Coulomb energy Δ*E*_ele_, and the entropic free energy Δ*G*_*s*_. The units of the (free) energies are *k*_*B*_ *T*.

### Examples of usage

#### Example 1: Basic calculations with the TBI server

**Fig 5:**
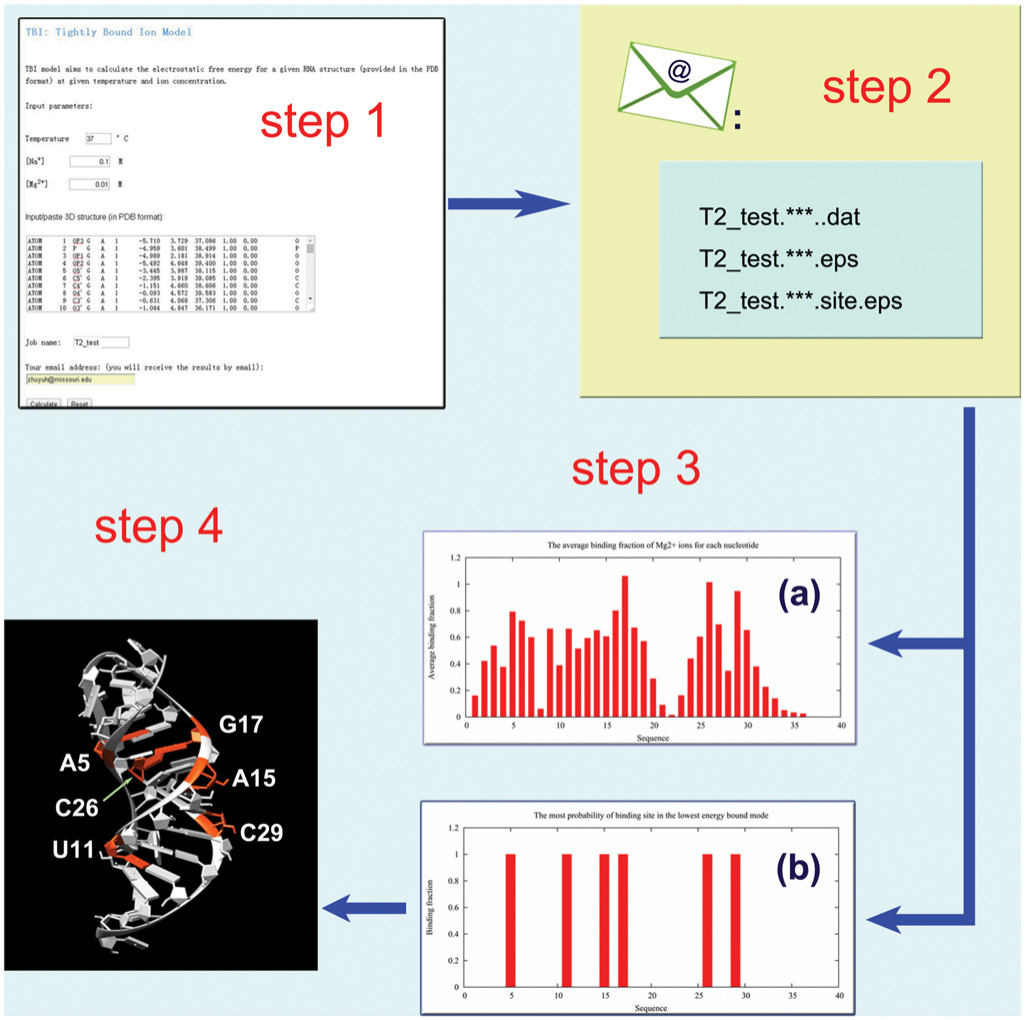
An example for the usage of the TBI server. The server computes the ion binding properties and the electrostatic free energies for T2 RNA (PDB code 2TPK [25]) in a solution with 10 mM Mg^2+^, 100 mM Na^+^, and *T* = 37°C.

Here, we show the basic usage of the TBI server through a simply example (shown in Fig. 5). The first step is to input the parameters and the RNA structure data (PDB code: 2TPK [25]) (see Fig. 5-step1). We then provide the job name and a valid email address if we choose to receive the results via email notification. We can simply leave the email address blank if we choose to retrieve the results from a webpage. In this example, we use “T2_test” for the job name. We then click “Calculate” to submit the job. The calculated results will be send to user provided email address (see Fig. 5 step2) or shown on the webpage for download when the calculations are finished. The three output files are “T2_test.*.dat”, “T2_test.*.eps” and “T2_test.*.site.eps” for this example. The “T2_test.*.dat” file contains all the free energy and ion binding properties data, which can be used to predict ion-dependent folding stabilities (see Examples 2 and 3 below). The two figures show the profile of the average binding fractions for all the nucleotides (see Fig. 3 or Fig. 5-step 3a) and the most probability binding sites (see Fig. 4 or Fig. 5-step 3b). Fig. 5-step 4 shows the most probable Mg^2+^ ion binding mode for the T2 RNA pseudoknot. It is important to note that the most probable binding mode does not necessarily correspond to the specific ion binding sites observed in the structure determination experiment. This is because the current TBI model does not account for effects that may be important for specific ion binding such as ion dehydration and ion chelated interactions with specific chemical groups.

#### Example 2: Mg^2+^-induced folding stability of T2 pseudoknot

**Fig 6:**
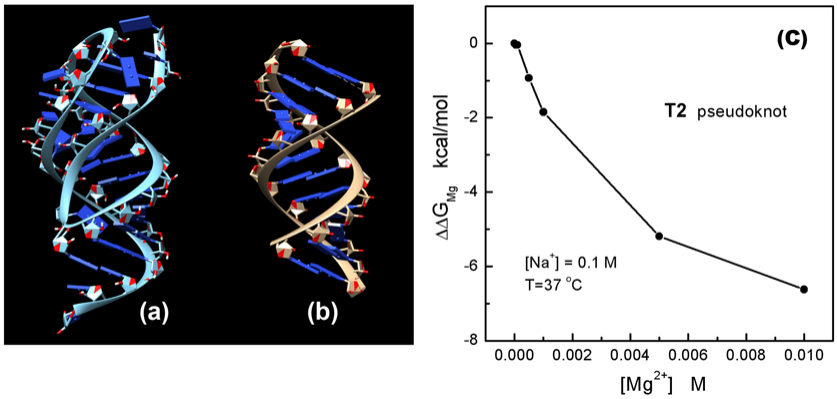
The Mg^2+^-induced folding stability ΔΔ*G*_Mg^2+^_ for the T2 pseudoknot. (a) and (b) show the 3D structures of T2 pseudoknot (the folded state) and the 24-nt A-form helix, respectively; (c) shows the Mg^2+^-induced folding stability.

To understand the ion-dependent folding stability of the T2 pseudoknot (see Example 1), we compute the ion-dependence of the free energy difference between the folded (pseudoknot) state and the intermediate (hairpin) state [19, 26, 27]. For the purpose of free energy calculation, previous studies suggested a computationally efficient method by using an effective 24-nt helix to represent the hairpin conformational ensemble [19, 26, 27]. We input the two structures (the effective helix and the pseudoknot; see Figs. 6ab) into the TBI server and compute the free energies at the different Mg^2+^ ion concentrations.

According to the thermodynamic cycle of RNA folding and [Mg^2+^] ion binding [26–29], we calculate the Mg^2+^-induced folding stability ΔΔ*G*_Mg^2+^_ from the following equation:

$$\begin{array}{c} \Delta \Delta G_{Mg^{2+}}=\Delta G_{Mg^{2+}}^{F}-\Delta G_{Mg^{2+}}^{I}=\left[\Delta G\left(F\cdot Mg^{2+}\right)-\Delta G_{o}^{F}\right]-\left[\Delta G\left(I\cdot Mg^{2+}\right)-\Delta G_{o}^{I}\right], \end{array}$$ (9)

where, $\Delta G_{{\text{Mg}}^{2+}}^{F}$ and $\Delta G_{{\text{Mg}}^{2+}}^{I}$ are the Mg^2+^-caused electrostatic free energy differences for the folded and the intermediate states [18, 26, 27], respectively, Δ*G*(*F* ⋅ Mg^2+^) and $\Delta G_{o}^{F}$ are the electrostatic free energies of the folded state with and without Mg^2+^ ions, respectively; Δ*G*(*I* ⋅ Mg^2+^) and $\Delta G_{o}^{I}$ are the electrostatic free energies of the unfolded state with and without Mg^2+^ ions, respectively. The electrostatic free energies Δ*G*(*F* ⋅ Mg^2+^) and $\Delta G_{o}^{F}$ for the folded state and Δ*G*(*I* ⋅ Mg^2+^) and $\Delta G_{o}^{I}$ for the intermediate state can be calculated from the TBI server. To estimate Δ*G*(*I* ⋅ Mg^2+^) and $\Delta G_{o}^{I}$, we use a 24-nt helix [18, 26, 27]: for the electrostatic free energy calculation:

$$\begin{array}{c} \Delta G\left(I\right)=\Delta G_{\mathrm{helix}}\cdot \frac{N_{I}}{N_{\mathrm{helix}}}=\Delta G_{\mathrm{helix}}\cdot \frac{36}{24} \end{array}$$ (10)

Here *N*_*I*_ (36) and *N*_helix_ (24) are the number of nucleotides of the T2 pseudoknot and the 24-nt helix, respectively. The calculated results are shown in the Table 2 and the curve of Mg^2+^-induced folding stability is shown in Fig. 6c.

**Table 2:** The table shows the calculated electrostatic free energies of the T2 pseudoknot (the folded state, Δ*G* ^*F*^), the 24-nt helix (Δ*G*_helix_) and the intermediate state (Δ*G* ^*I*^) under various [Mg^2+^].

[Mg^2+^] (in mol)	0	0.00005	0.0001	0.0005	0.001	0.005	0.01
Δ*G* ^*F*^ (in kcal/mol)	59.52	59.47	59.45	58.41	57.27	51.64	47.28
Δ*G*_helix_ (in kcal/mol)	30.30	30.29	30.28	30.18	30.04	28.51	26.55
Δ*G* ^*I*^ (in kcal/mol)	45.45	45.43	45.42	45.26	45.05	42.77	39.82

#### Example 3: Estimation of Mg^2+^ ion uptake

The uptake of ions is the increase in the number of bound ions in a process such as RNA structural change [30, 31]. The TBI server gives the average binding fraction of monovalent and divalent ions for a given RNA structure. The average uptake Mg^2+^ ions for the folding process can be estimated from the difference in binding fraction between the folded and the unfolded RNA [21, 30, 31]. Using T2 pseudoknot as an example, we estimate the Mg^2+^ uptake for the folding from the hairpin intermediate to the final pseudoknot state.

**Fig 7:**
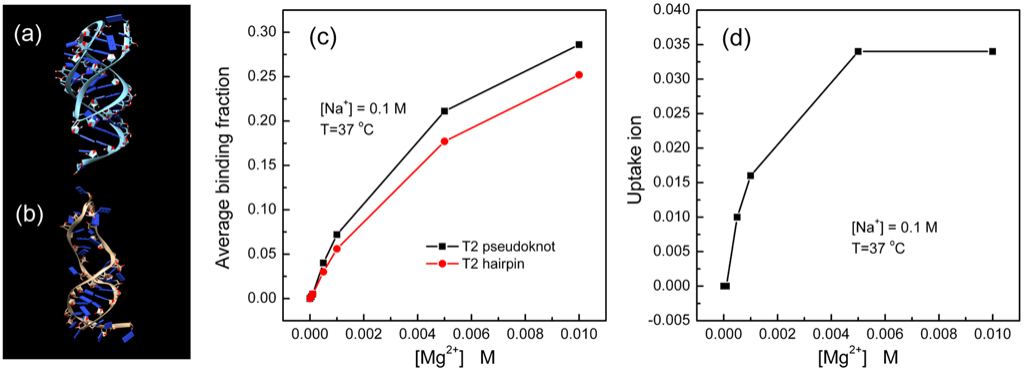
The binding fraction and the uptake of Mg^2+^ ions as a function of [Mg^2+^]. (a) and (b) show the 3D structures of the T2 pseudoknot (the folded state) and T2 hairpin (the intermediate state), respectively; (c) shows the binding fraction curves for the T2 pseudoknot (black) and the hairpin (red). (d) shows the Mg^2+^ uptake as a function of [Mg^2+^].

Since the ion binding distribution can be sensitive to the RNA structure, we need to generate explicitly the ensemble of discrete hairpin conformations [32–35]. This can be achieved by using a separate method such as molecular dynamics or Monte Carlo simulational method or a coarse-grained conformational sampling method. To the purpose of illustrate the electrostatic calculations, we use the lowest free energy T2 hairpin structure as an example. The structure is based on a conformational ensemble generated by molecular dynamics simulation [34, 35]. Using the TBI server, for the different Mg^2+^ ion concentrations, we can compute the Mg^2+^ ion binding fractions (*f*_Mg^2+^_) for the T2 pseudoknot and the hairpin, respectively. We then predict the Mg^2+^ ion uptake in the folding process from the hairpin to the pseudoknot. As shown in Fig. 7, with a fixed 0.1M Na^+^ and increasing [Mg^2+^] from 0 M to 5 mM, the Mg^2+^ ion uptake increases from 0 to 0.035 per nucleotide. Table 3 and Fig. 7 show the TBI-calculated binding fractions and the Mg^2+^ ion uptake curve as a function of [Mg^2+^].

**Table 3:** The table lists the calculated average binding fraction of the T2 pseudoknot (the folded state, *f* ^*F*^) and the intermediate state (*f* ^*I*^) under the different [Mg^2+^] conditions.

[Mg^2+^] (in mol)	0	0.00005	0.0001	0.0005	0.001	0.005	0.01
*f* ^*F*^	0	0.002	0.005	0.040	0.072	0.211	0.286
*f* ^*I*^	0	0.002	0.005	0.030	0.056	0.177	0.252

## Conclusion

Accurate prediction of ion-mediated forces in the stabilization of RNA structure is critical to understanding RNA structure and function. Multivalent ions, especially Mg^2+^ ions, are crucial for RNA tertiary structure folding. For the multivalent ions, ion correlation and fluctuation may play a important role in RNA folding. This demands a web server that can treat ion correlation and fluctuation effects in RNA folding. TBI is such a server. For a user provided RNA structure and ionic condition, the server computes ion binding properties, electrostatic free energies and various components. The TBI server provides results can be used to predict the ion-dependence of folding stability and ion uptake/release in the folding process. In the future development, we plan (a) to expand the server to treat other types of ions beyond the Mg^2+^ and Na^+^ ions, (b) to use a more detailed charge distribution (such as partial charge) to model RNA charges, and (c) to include ion dehydration effects for ion binding.
